# Narrative foreclosure among patients with chronic diseases: a scoping review

**DOI:** 10.3389/fpubh.2025.1735227

**Published:** 2026-01-06

**Authors:** Zhounan Xu, Shiya Cui, Nana Hu, Manyao Sun, Xueying Huang, Kaiyue Chen, Shilei Wang, Yuyu Jiang

**Affiliations:** 1 Research Office of Chronic Disease Management and Rehabilitation, Department of Nursing, Wuxi School of Medicine, Jiangnan University, Wuxi, Jiangsu Province, China; 2 School of Nursing, Wuxi Taihu University, Wuxi, Jiangsu Province, China

**Keywords:** associated factors, chronic disease, clinical manifestations, conception, intervention strategies, narrative foreclosure, scoping review

## Abstract

**Aims:**

To comprehensively integrate existing research on narrative foreclosure among patients with chronic diseases, aiming to explore how the concept is described and whether its clinical manifestations differ across various disease contexts, as well as to summarize the associated factors and intervention strategies.

**Design:**

Scoping review.

**Data sources:**

Systematic searches were performed in seven databases (CNKI, Embase, PubMed, CINAHL, PsycINFO, Scopus, and Web of Science) from their inception until March 2025.

**Methods:**

We followed the framework proposed by Arksey, O'Malley, and Levac. The review was reported in accordance with the Preferred Reporting Items for Systematic Reviews and Meta-Analyses Extension. Research on narrative foreclosure in patients with chronic diseases was included, regardless of the specific setting in which it was conducted.

**Results:**

A total of nine studies were included, covering both cancer and other chronic diseases. The primary outcomes are as follows. The core concept of narrative foreclosure in the context of chronic diseases refers to a state in which individuals believe their life story has ended; however, its specific descriptions vary across chronic diseases such as asthma and depression. The clinical manifestations include negative emotions and maladaptive behaviors. Twelve associated factors were identified, including age, psychological factors (such as ego integrity and hope), social support, and educational level. Interventions targeting narrative foreclosure involved the application of hope theory, dignity therapy, meaning-centered approaches, and narrative nursing.

**Conclusion:**

At present, research on narrative foreclosure in the field of chronic diseases is limited. Narrative foreclosure has been proven to have adverse effects on disease management among patients with chronic diseases. Therefore, it is particularly important to pay attention to the clinical manifestations of patients' narrative foreclosure and to identify the factors that promote or hinder it. Additionally, existing intervention measures need further verification, and the concepts of positive psychology should be integrated into their design and implementation.

**Registration number:**

Our review protocol was prospectively registered on the Open Science Framework (OSF). Unique Identifier: 10.17605/OSF.IO/5RQKM.

## 1 Introduction

Globally, approximately half of all adults suffer from at least one chronic disease (1), and by 2050, this phenomenon is expected to become even more prevalent among people aged 50 and above (2). Chronic diseases have become the leading cause of death worldwide (3), and their economic burden continues to grow, with total related costs projected to reach $47 trillion by 2030 (4). Unlike acute diseases, chronic diseases are characterized by a prolonged state of “living with disease,” in which patients find it difficult to return to their pre-illness health and cannot clearly predict the endpoint of their disease course (5). This uncertainty exposes patients not only to persistent physical symptoms but also to psychological comorbidities such as anxiety and depression (6). Over time, patients may enter a state of diminished vitality, perceiving their fate as constrained by illness and their lives as ended and meaningless (7). American scholar Mark Freeman has defined this phenomenon as “narrative foreclosure” (NF) (8).

Narrative foreclosure refers to a state in which individuals believe their life story has ended, including the end of past experiences and the end of future experiences. Specifically, the end of past experiences refers to an individual's inability to reinterpret and give meaning to past experiences from a new perspective. The end of the future refers to an individual no longer making commitments or having any anticipations for the future (8, 9). Individuals in a state of narrative foreclosure experience a form of life cessation that precedes biological death, as they lose their agency the capacity to generate meaning and initiate action (10). As Benjamin Franklin once said, “*Some people die at 25, but are not buried until 75.”* Research indicates that chronic patients experiencing narrative foreclosure often suffer from intense psychological distress, such as depression and hopelessness (11), and tend to adopt maladaptive health behaviors, including refusal to participate in rehabilitation programs (12) or even suicidal actions (13). Therefore, narrative foreclosure can reduce patients' proactive participation in chronic disease management, and the resulting psychological and behavioral effects may negatively influence both quality of life and clinical prognosis. It is imperative that this issue receives increased attention in the healthcare field.

Academic discussions of narrative foreclosure can be traced back to literary studies. Russian literary scholar Morson, in his *Narrative and Freedom*, proposed the concept of “epilog time” (14), which is conceptually similar to what Mark Freeman later described as narrative foreclosure. Subsequently, this concept was introduced into the field of gerontology, exemplified by McCullough's description of “aging stagnation,” in which individuals appear trapped in an inescapable past, losing anticipation and imagination for the future (15). In recent years, the scope of narrative foreclosure research has gradually expanded into medical contexts, particularly in relation to chronic diseases such as cancer and asthma (13, 16).

At present, research on narrative foreclosure in the context of chronic diseases remains fragmented, and there is a lack of systematic reviews specifically synthesizing findings for patients with chronic diseases. Existing reviews have largely focused on healthy populations. Based on the manifestations of narrative foreclosure among healthy individuals, Freeman identified four types: the “*dead end,”* the “*point of no return,”* the “*narrative of regret,”* and the “*blacker rapidly.”* (17) Building on this framework, Yang et al. further classified narrative foreclosure into occupational, traumatic, doubt-related, and aging-related types according to its underlying causes (10). However, these studies do not fully reflect the unique narrative dilemmas faced by patients with chronic diseases.

Therefore, this study aims to synthesize existing research on narrative foreclosure among patients with chronic diseases, to examine how the concept has been described across different disease contexts, to summarize its clinical manifestations, associated factors, and intervention strategies, and to identify current research gaps, thereby providing direction for future investigations.

## 2 Method

This scoping review was conducted in accordance with the methodological framework proposed by Arksey and O'Malley (18). It included refinements based on recommendations by Levac et al. to enhance the rigor and comprehensiveness of the identification and synthesis process (19). The reporting of this review adhered strictly to the Preferred Reporting Items for Systematic Reviews and Meta-Analyses Extension for Scoping Reviews (PRISMA-ScR) guidelines, with the completed checklist provided in Appendix S1 (20). Our protocol is registered in the Open Science Framework registry (https://doi.org/10.17605/OSF.IO/5RQKM).

### 2.1 Identifying the research question

This review aimed to explore the literature on narrative foreclosure among patients with chronic diseases. The primary research questions were refined to ensure comprehensive coverage of the available literature, as follows:

a. Are there differences in how the concept of narrative foreclosure is described across different chronic disease contexts?
b. Are there differences in the clinical manifestations of narrative foreclosure among patients across different chronic disease contexts?
c. What are its associated factors?
d. What intervention strategies have been reported to address this phenomenon?

### 2.2 Identifying relevant studies

We searched seven databases (CNKI, Embase, PubMed, CINAHL, PsycINFO, Scopus, and Web of Science) from their inception to March 2025 using “narrative foreclosure” and related free-text terms (e.g., “end of story,” “story closure”) connected with the Boolean operator OR, with the search limited to publications in Chinese and English. The detailed search strategy is provided in Appendix S2. All records retrieved from the databases were imported into Zotero for de-duplication, after which two reviewers independently screened the full texts according to the inclusion and exclusion criteria. Any disagreements were resolved through discussion or, when necessary, adjudication by a third reviewer, and the reasons for exclusion at the full-text screening stage were documented.

### 2.3 Study selection

#### 2.3.1 Inclusion criteria

(1) Participants: Studies involving individuals diagnosed with chronic diseases. [Note: We adopted a relatively broad operational definition of “chronic disease,” consistent with major public health frameworks such as those of the World Health Organization and the United States Department of Health and Human Services (HHS), which define chronic diseases as conditions that last a year or more and require ongoing medical attention and/or limit activities of daily living (21)].

(2) Concept: Narrative foreclosure refers to a state in which individuals believe their life story has ended, including the end of past experiences and the end of future experiences. Specifically, the end of past experiences refers to an individual's inability to reinterpret and give meaning to past experiences from a new perspective. The end of the future refers to an individual no longer making commitments or having any anticipations for the future.

(3) Context: Studies conducted in any chronic disease context were considered eligible. Study settings included community, family, long-term care facilities, and healthcare or rehabilitation settings. Study designs comprised cross-sectional studies, case reports, qualitative conceptual studies, and qualitative research.

#### 2.3.2 Exclusion criteria

Studies were excluded if they:

(1) Focused exclusively on healthy populations;

(2) Were published in languages other than Chinese or English;

(3) Were theses, conference papers, books, book chapters, gray literature, or articles without accessible full text;

(4) Were unpublished or duplicate publications.

### 2.4 Charting the data

Two reviewers independently extracted key data from each included study using Microsoft Excel. Extracted items included: authors, publication year, country, methodology, study design, type of chronic disease, specific descriptions, clinical manifestations, associated factors, and intervention strategies. Discrepancies in data extraction were resolved through discussion. If disagreement persisted, a third reviewer was consulted. No quality appraisal of included studies was undertaken.

### 2.5 Collating, summarizing, and reporting the results

First, descriptive statistics (frequency and range) were used to summarize key characteristics of the included studies, including authors, publication year, country, methodology, study design, and type of chronic disease. Subsequently, through descriptive summaries and inductive analysis, the specific descriptions, clinical manifestations, associated factors, and intervention strategies of narrative foreclosure in the chronic disease context were integrated according to the research questions. Descriptive methods were employed to illustrate the specific descriptions of narrative foreclosure across various chronic disease types. Tables were used to present the clinical manifestations of narrative foreclosure across different chronic disease types, the associated factors across different dimensions, and the intervention strategies from different studies. This approach reveals both cross-disease commonalities and contextual variations.

## 3 Results

### 3.1 Literature search

**Figure 1:**
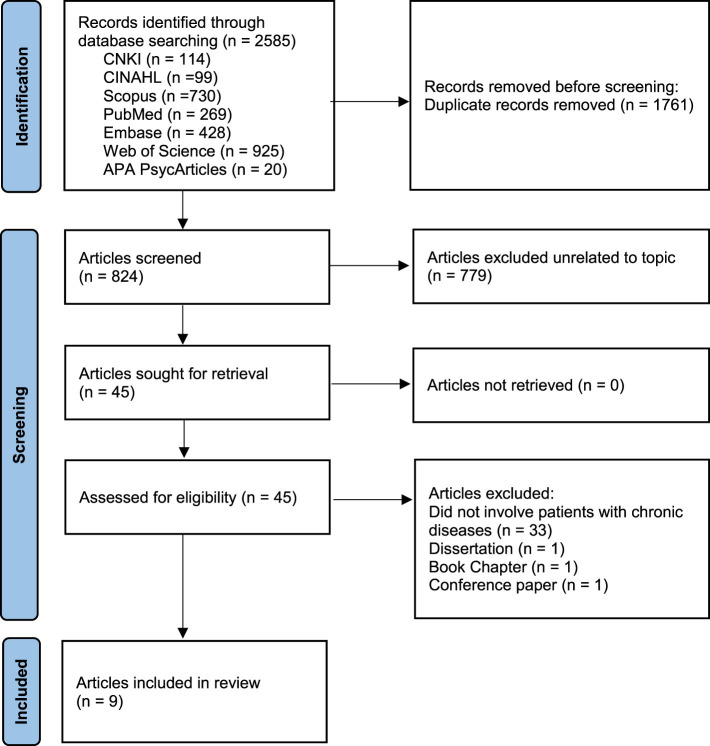
Literature screening process.

The detailed process of study selection is illustrated in Figure 1. An initial total of 2,585 records were identified using Zotero. After removing 1,761 duplicates, 779 articles were excluded based on titles and abstracts for not addressing the concept of narrative foreclosure. Upon full-text screening, 33 studies were further excluded for not involving patients with chronic diseases. Additionally, one dissertation, one book chapter, and one conference paper were excluded. Ultimately, 9 studies were included in this scoping review.

### 3.2 Characteristics of included studies

The included studies were published between 2007 and 2024. The distribution by publication year is presented, with the majority published in 2023 (*n* = 2) and 2014 (*n* = 2). The remaining studies were published in 2007 (*n* = 1), 2011 (*n* = 1), 2018 (*n* = 1), 2022 (*n* = 1), and 2024 (*n* = 1). The studies originated from six countries: China (*n* = 2), the Netherlands (*n* = 2), and England (*n* = 2); the remaining studies were from Canada (*n* = 1), Sweden (*n* = 1), and the United States (*n* = 1). Study designs varied across the included literature and comprised qualitative conceptual studies (*n* = 2), qualitative studies (*n* = 3), cross-sectional studies (*n* = 2), and case reports (*n* = 2). A wide range of chronic disease types were involved. Cancers included stomach cancer (*n* = 1), pancreatic cancer (*n* = 1), and duodenal cancer (*n* = 1). Other diseases included chronic diseases secondary to severe brain injury (*n* = 1), depression (*n* = 2), asthma (*n* = 1), dementia (*n* = 1), and secondary hyperparathyroidism secondary to chronic kidney disease (*n* = 1). More detailed information can be found in Table 1.

**Table 1:** The basic information of the included articles.

**Authors**	**Published**	**Country**	**Methodology**	**Study design**	**Type of chronic disease**
Bohlmeijer et al. (9)	2011	Netherlands	Theoretical analysis method and case analysis method	Qualitative Conceptual Study	Stomach Cancer
Antelius (12)	2007	Sweden	Observation and interviews	Qualitative Study	Chronic disease resulting from severe brain injury (such as multiple sclerosis and Huntington's disease)
Bohlmeijer et al. (25)	2014	Netherlands	Quantitative scale development and validation methodology	Cross-Sectional Study	Depression
Griffin et al. (16)	2014	England	Narrative inquiry and interpretive ethnography	Qualitative Study	Asthma
Witham et al. (23)	2018	England	Theoretical analysis method and case analysis method	Qualitative Conceptual Study	Dementia
Weng et al. (11)	2022	United States	Case analysis method	Case Report	Pancreatic Cancer
Randall (22)	2023	Canada	Theoretical analysis method	Qualitative Study	Depression
Yin (24)	2024	China	Quantitative scale development and psychometric reliability-validity assessment methodology	Cross-Sectional Study	Secondary hyperparathyroidism secondary to chronic kidney disease
Li et al. (13)	2023	China	Case analysis method	Case Report	Duodenal Cancer

### 3.3 Specific descriptions of narrative foreclosure in different chronic diseases

Although the included studies consistently define narrative foreclosure as a state in which individuals believe their life story has ended (9, 11–13, 22–24), there are some differences in how it is described in chronic diseases such as asthma and depression.

#### 3.3.1 Trapped in an unimaginative single story

In a qualitative study of women with asthma learning to run, narrative foreclosure was described as individuals becoming trapped within an unimaginative single story (16). Patients framed their life story plots as being “too old, physically inadequate, or unsuited for exercise,” internalizing sociocultural notions that aging equates to decline and that women's bodies have inherent limitations. This constrained their ability to find meaning in life and envision future possibilities.

#### 3.3.2 Stagnation of identity development

In a cross-sectional study involving patients with depression, narrative foreclosure was described as a state of stagnation in personal identity development (25). Individuals became confined to a single, fixed self-identity and were unable to engage in diverse identity exploration or reconstruction. This stagnation limited their ability to reinterpret past experiences and to view the future positively, thereby impeding psychological adaptation and growth.

### 3.4 Clinical manifestations of narrative foreclosure in patients with different chronic diseases

Among the included studies, only five reported the clinical manifestations of narrative foreclosure in patients with chronic diseases. Overall, narrative foreclosure in chronic disease patients typically manifests clinically as negative emotions and maladaptive behaviors. The specific manifestations across different disease types are summarized in Table 2.

**Table 2:** Clinical manifestations of narrative foreclosure in patients with different chronic diseases.

**Disease type**	**Negative emotions**	**Maladaptive behaviors**	**Summary**
Stomach Cancer (9)	dejection, loneliness, hopelessness	Stopping work; alcohol abuse; engaging in nightlife	Negative emotions and maladaptive behaviors are both pronounced.
Duodenal cancer (13)	Feelings of inferiority and timidity in the presence of offspring, and irritability or irrationality toward one's spouse.	Avoidance of family communication; suicidal behaviors	Emotional expression differs significantly depending on the type of close relationship
Pancreatic cancer (11)	Hopelessness, dejection, and worry	No maladaptive behaviors; continues to follow treatment	Negative emotions are pronounced.
Brain injury (12)	Feelings of loss and anger	Doubts about and resistance to rehabilitation training	Negative emotions and maladaptive behaviors are both pronounced.
Asthma (16)	No significant mood depression	Long-term avoidance of running or other physical activities	Maladaptive behaviors are pronounced.

### 3.5 Factors associated with narrative foreclosure in patients with chronic diseases

Among the 9 studies, only 8 examined factors associated with narrative foreclosure among chronic disease patients. Two studies used scales to quantitatively measure associated factors. One study set narrative foreclosure as the dependent variable and selected 174 patients with mild to moderate depression (25). The results showed that on the “future” dimension of the Narrative Foreclosure Scale (NFS), age (*p* < 0.001) and depression severity (*p* < 0.01) were positively correlated, while ego integrity (*p* < 0.001), positive mental health (*p* < 0.001), and growth motivation (*p* < 0.001) were negatively correlated. In addition, educational level (*p* < 0.001), ego integrity (*p* < 0.001), and positive mental health (*p* < 0.001) were negatively correlated with the “past” dimension of the NFS, whereas depression severity (*p* < 0.001) and rumination (*p* < 0.001) were positively correlated with this “past” dimension.

Another study treated narrative foreclosure as a latent psychological variable and, through the localization and reliability and validity testing of the scale, confirmed a significant positive correlation between it and stigma (*p* < 0.01) (24).

The remaining six studies conducted a descriptive exploration of factors that may be associated with patients' narrative foreclosure. The factors involved included age (*n* = 3) (9, 16, 22), hope (*n* = 1) (12), social support (*n* = 3) (9, 13, 23), negative societal stereotypes related to aging (*n* = 3) (9, 16, 22) and dementia (*n* = 1) (23), and the biomedical model based on “disease management” (*n* = 1) (23).

In summary, a total of 12 factors associated with narrative foreclosure in patients with chronic diseases were identified. The identified factors were systematically synthesized and are detailed in Table 3.

**Table 3:** Factors associated with narrative foreclosure in patients with chronic diseases.

**Dimension**	**Factors**
Individual trait factors	Age (9, 16, 22, 25)
Psychological factors	Depression (25); Ego integrity (25); Positive mental health (25); Hope (12); Rumination (25); Growth motivation (25); Stigma (24)
Interpersonal network factors	Social support (9, 13, 23)
Living and working condition factors	Education level (25)
Policy environment factors	Negative societal stereotypes (9, 16, 22, 23); A biomedical model based on “disease management” (23)

### 3.6 Interventions for narrative foreclosure in patients with chronic diseases

Among the 9 included studies, 7 (78%) proposed potential interventions for addressing narrative foreclosure in patients with chronic diseases. However, none of these studies conducted quantitative evaluations of the interventions, and all were implemented in offline, in-person settings (see Table 4 for details). Two of the seven studies described strategies for applying narrative nursing (13, 23). Another qualitative study involving patients with severe brain injury emphasized the value of setting achievable short-term goals, which could help sustain a sense of hope through feelings of short-term accomplishment, thereby disrupting the sense of closure toward the future (12). A case report discussed the use of Dignity Therapy (DT) and Meaning-Centered Psychotherapy (MCP) for patients with advanced cancer (11). These approaches were found to alleviate psychological distress, enhance a sense of dignity, and reinforce patients' sense of life purpose and meaning (11). One qualitative research advocated for a shift in the narrative perspective from “tragedy” to “adventure”, emphasizing themes such as discovery, exploration, and personal growth (22). Among the seven studies mentioning interventions, two studies recommended Life Review Therapy, but only offered preliminary suggestions without elaborating on specific intervention details (9, 25).

**Table 4:** Interventions for narrative foreclosure in patients with chronic diseases.

**Studies**	**Intervention name**	**Specific content**
Antelius (12)	Narrative integration with hope	1. Setting Small Goals: Goals should be clearly defined, immediate, and achievable. Typically, these are small, short-term intermediate objectives closely integrated with the patient's daily training routines. 2. Motivational Mechanism: Healthcare providers visualize patients' completion of small goals as “incentives” to motivate and encourage active engagement in rehabilitation.
Witham et al. (23)	Narrative nursing	1. Construction of Counterstories: By attentively listening to patients' fragmented expressions, nurses can assist in renaming and reconstructing these narratives, helping patients resist marginalization and silence associated with stigmatized identities. 2. Valuing Small Stories: Nurses should foster everyday conversations by actively listening to and paying attention to patients' “small stories,” including habits, emotions, or nonverbal cues, providing ongoing support throughout.
Li et al. (13)	Narrative nursing	1. Narrative Reconstruction: Regular face-to-face conversations guide patients to share life experiences, such as family background and past careers, as well as their views and feelings about disease and death, helping them assign new, positive meanings to these events. 2. Family Relationship Repair and Support: Encourage family visits and facilitate communication between patients and their relatives. 3. Rebuilding Social Value (Introducing External Witnesses): Organize online patient support groups where patients can share expertise in specific areas (e.g., plant care), enabling them to rediscover a sense of purpose and value beyond their disease.
Weng et al. (11)	Dignity therapy	1. Preparation: Provide participants with a list of interview questions in advance (e.g., life history, moments when they felt most alive, what they hope their family remembers), allowing them to reflect and identify topics they wish to discuss during formal interviews. 2. Interviews: Patients engage in two to three interview sessions with trained therapists. 3. Document Generation: Interview content is transcribed and edited for clarity and coherence, producing a “Generativity Document” (GD). 4. Document Confirmation and Sharing: The document is reviewed and confirmed by participants, with further editing as needed to ensure completeness. Participants then decide whether to share it with others.
	Meaning-centered psychotherapy	1. Therapeutic Formats: Both group and individual therapy formats are utilized. 2. Therapy Duration: Typically conducted once weekly over a course of 7 to 8 sessions.
Randall (22)	Adventure therapy	1. Adventure Outward: Stepping beyond the comfort zone through activities such as traveling, learning new skills, or engaging in social events (e.g., the 80-year-old wingsuit flier Tom Lackey). 2. Adventure Inward: Achieving spiritual growth via self-reflection and inner exploration (e.g., Jung's concept of “turning inward”), counteracting anxiety related to aging. 3. Adventure Backward: Integrating past experiences through autobiographical writing or reminiscence, fostering life coherence. 4. Adventure Forward: Confronting death and the unknown by drawing on Near-Death Experiences (NDEs) and Gerotranscendence theory, viewing death as a transition rather than an end.

## 4 Discussion

### 4.1 Summary of key findings

This scoping review is the first to systematically summarize existing research on narrative foreclosure in patients with chronic diseases from four key perspectives: conceptual descriptions, clinical manifestations, associated factors, and intervention strategies. First, within the context of chronic diseases, the core concept of narrative foreclosure refers to a state in which individuals believe their life story has ended. However, its specific descriptions vary across disease contexts, with notable differences observed in conditions such as asthma and depression. Subsequently, this review analyzed the clinical manifestations of narrative foreclosure among patients across different chronic disease contexts. In addition, twelve associated factors were identified. Finally, this review analyzes and summarizes both the intervention strategies proposed by scholars in the included studies and the narrative care practices implemented in case reports. These findings aim to inform future research in designing effective intervention strategies and practical approaches to help break narrative foreclosure in patients with chronic disease.

### 4.2 The core concept of narrative foreclosure

Although the specific description of narrative foreclosure varies across different chronic diseases, its core concept consistently points to a state in which individuals believe their life story has ended. In this state, patients are no longer able or willing to continue constructing and developing their life narratives. This is not attributable to a single cause, but rather to the interplay of physiological (somatic symptoms) (4), psychological (mental health problems) (26), and sociocultural (aging equals decline) factors (16). Postmodern narrative psychotherapy posits that the problem does not lie within the patient, but rather in the dominant “illness story,” which suppresses alternative possible stories (27). It advocates addressing the predicament of story finality by reconstructing life meaning and empowering patients to re-author their own stories (27). Research suggests that the long-term, irreversible, and recurrent nature of chronic diseases creates persistent physical and psychological constraints, fostering a dominant, negative, and one-dimensional story (e.g., patients frequently voice anchor statements such as “I'll never get better” or “My life has lost meaning”) (28, 29). A prominent theme in stories constructed to break out of narrative foreclosure is redemption (30). Redemption narratives enable patients to reconstruct meaning and experience personal growth amid adversity, leading to a positive resolution in which adversity yields beneficial outcomes (30). Importantly, meaning reconstruction is not a denial of the disease but a process of situating the illness story within a larger life story, thereby reestablishing patients' life stories and agency (31). Future research and clinical practice should gain in-depth insight into patients' subjective experiences and integrate meaning-centered, empowerment-based interventions throughout the entire course of treatment, enabling patients to take an active part in medical decision-making and adopt healthy behaviors. A deeper understanding of narrative foreclosure will inform the development of humanistic care techniques, thereby driving innovation in chronic disease management models.

### 4.3 Causes of clinical manifestations of narrative foreclosure in patients with chronic diseases

This study found that chronic disease patients experiencing narrative foreclosure display a range of negative emotions and maladaptive behaviors. These emotions arise because narrative foreclosure erodes their capacity to construct meaning in life. According to life-meaning theory (32), human beings possess an intrinsic need to seek life meaning. When this need remains chronically unmet, individuals fall into distress and consequently exhibit negative emotions such as depression, despair, and low self-worth (33). Previous research indicates that these negative emotions contribute to poor patient outcomes (34). Maladaptive behaviors appear to stem from a deficit of hope in patients (12). Hope is conceptualized as an inner force that drives life engagement and strengthens patients' ability to endure prolonged treatment (35). Research indicates that hope deficient patients frequently demonstrate treatment avoidance (12), disengagement from shared decision-making (36), and reduced adherence to prescribed therapies (37). The World Health Organization has highlighted that adherence-related behaviors can exert a greater influence on health outcomes than individual medication regimens (38). The negative emotions, passive health behaviors, and reduced decision-making participation triggered by narrative foreclosure are precisely the core domains that require patients' self-management (39). Research indicates that self-management interventions constitute a key element of chronic disease care (40). Consequently, narrative foreclosure may undermine chronic disease management outcomes and contribute to greater consumption of healthcare resources. Moreover, a Chinese case report noted differential affective expression toward children vs. spouses (13), which appears to reflect the hierarchical prioritization of family ties characteristic of some traditional Chinese families. In some Chinese families adhering to traditional patterns, the vertical parent–child axis often takes precedence over the horizontal spousal axis (41). This relational hierarchy shapes differentiated role expectations and patterns of emotional expression among family members. The profound love individuals feel for their children, characterized by strong altruism and a sense of continuity, often leads patients to conceal vulnerability and maintain authority in front of their offspring, resulting in restrained and controlled emotional displays (42). In contrast, within a spousal relationship based on egalitarian partnership, individuals are more likely to regard their spouse as a “safe haven” for emotional release and psychological support, leading to more overt and, at times, negative emotional expressions (43). Future research can further explore the interactive mechanisms between cultural background and narrative foreclosure. Clinically, assessments and understandings of patients with narrative foreclosure need to be conducted from a cultural perspective in order to deliver truly personalized and precise narrative interventions.

### 4.4 Factors associated with narrative foreclosure in patients with chronic diseases

After systematically reviewing the included studies, the factors associated with patients' narrative foreclosure can be categorized into facilitators and barriers. Among the facilitators, this study identified that higher educational attainment and sufficient social support together constitute key internal and external resources that help patients resist narrative foreclosure (9, 13, 23, 25). Similarly, patients with high levels of ego integrity, hope, and growth motivation tend to exhibit lower levels of narrative foreclosure (25). Ego integrity enables patients to accept their disease experiences and assign positive meaning to them (44). Hope embodies a proactive attitude and positive expectations for the future (45). Growth motivation drives patients to set meaningful future-oriented goals and engage in new activities (46). Conversely, among the barriers, societal stereotypes such as “aging equals decline” and “patients with dementia can only await death” are often internalized by patients through their daily experiences and self-reflection as disease-related stigma (9, 16, 22, 23). This internalization reinforces self-negation, shrinks the imaginative space for the future, and accelerates the formation of narrative foreclosure (9, 16, 22, 23). Additionally, depression, commonly accompanied by a loss of meaning and excessive focus on negative disease-related information, limits patients' capacity to envision future possibilities and is a significant predictor of narrative foreclosure (25). This study also found that rumination drives patients to continuously focus on their disease and the negative emotions it provokes, thereby deepening the degree of narrative foreclosure to some extent (25). However, rumination is not entirely detrimental. Treynor et al. proposed a two-dimensional model distinguishing between reflective pondering and brooding (47). Reflective pondering refers to an individual's constructive reflection on negative events or emotions, aiming to understand their nature, causes, and potential solutions (48). This form of rumination allows individuals to move beyond the negative event itself and maintain an open attitude toward future development (48). In contrast, brooding involves repetitive dwelling on personal failures and painful experiences without actively seeking solutions or learning from them (49). This maladaptive rumination traps individuals in negative emotions and fosters a sense of hopelessness (50). Going forward, longitudinal cohort studies should continuously track the dynamic changes of these facilitating and hindering factors to clarify their causal effects on patients' narrative foreclosure. Furthermore, based on the multidimensional indicators identified in this review, databases can be established and predictive models developed using machine learning algorithms. Through data-driven precise identification and targeted interventions, it is hoped that the formation of narrative foreclosure in chronic disease patients can be delayed or even reversed, ultimately improving their quality of life and effectiveness in self-managing their disease.

### 4.5 Interventions for narrative foreclosure in patients with chronic diseases

Current interventions addressing narrative foreclosure in chronic disease patients involve applications of hope, dignity, and meaning-in-life theories (11, 12), as well as narrative nursing (13, 23). However, there remains a notable lack of randomized controlled trials to quantitatively evaluate their effectiveness. In times of physical and psychological crisis, spirituality can provide hope, strength, peace, and resilience (51). A multicenter cross-sectional study has also shown that spiritual health is closely related to quality of life (52). Spiritual interventions may have potential benefits for patients experiencing narrative foreclosure. In the included studies, one case study of cancer patients has provided a detailed account of the entire process by which healthcare providers reshaped patients' disease narratives through listening to their stories, employing meaning reconstruction techniques, and introducing “external witnesses” (13). The key to breaking narrative foreclosure lies in artistically reconstructing the plot of the patient's story and encouraging the patient to put the plot into practice through concrete actions, thereby gaining a new interpretation of the original story and experiencing new positive meanings. This process poses significant challenges for healthcare providers. It is worth emphasizing that narrative interventions that remain purely at the discursive level can only prompt patients to discover their own latent resources and provide new interpretive perspectives on their disease experiences (53). Only by further transforming “discursive narratives” into “action narratives”, enabling patients to gain dual physical and emotional experiences through practice in real-life contexts, can effective health behavior changes be stimulated and sustained long-term. Additionally, the cultural tendencies underlying narrative foreclosure should be examined, and intervention models with local cultural characteristics should be explored. The current healthcare system focuses on the “symptom trajectory” of diseases while neglecting patients' “life stories”, forcing patients to slip from being active participants to passive sufferers of disease (54). Future research should include a large number of quantitative studies to develop a new narrative-based model for chronic disease management. With the growing application of contemporary artificial intelligence (AI) technologies in healthcare (57), we anticipate future integrations between AI and narrative approaches—helping patients shift from being merely “objects of documentation” to becoming active authors of their own stories, allowing technological innovation and the humanities of medicine to enrich one another within a narrative that never ends. Park et al. found that conversational AI chatbots can guide users to revisit key life events, clarify emotions and thematic cues, and foster deeper self-reflection through situated prompts, thereby supporting the construction of more coherent and meaningful personal narratives (55). A systematic review has further shown that AI-powered chatbots demonstrate good feasibility and high acceptability in supporting self-management and psychosocial well-being among patients with chronic diseases (56).

## 5 Strengths and limitations

This study elucidates the core concept of narrative foreclosure and synthesizes the clinical manifestations, associated factors, and intervention strategies related to narrative foreclosure in patients with chronic diseases. This not only enhances understanding of narrative foreclosure but also provides clinicians with a novel perspective to deepen their recognition of the psychological issues and adverse health behaviors in this patient population.

This review did not include a quality assessment or bias evaluation of the included studies. Therefore, it primarily describes the distribution of evidence. It cannot support causal inferences. Additionally, the search was limited to Chinese and English literature. This introduces potential language bias and possibly overlooks important studies published in other languages. Future research should expand to multi-language searches and, where feasible, complement findings with systematic reviews or meta-analyses to enhance the reliability and generalizability of the evidence.

## 6 Conclusion

Research on narrative foreclosure among chronic disease patients remains in its early stages, with fragmented evidence and limited overall scale. Narrative foreclosure undermines patients' ability to construct meaningful interpretations of their disease experiences, thereby impairing treatment adherence, social participation, and overall quality of life. It represents a subtle yet easily overlooked risk factor in long-term chronic disease management. If left unrecognized and unaddressed, the resilience that patients build to coexist with their disease will be steadily eroded. To effectively mitigate this issue, researchers must clarify clinical manifestations of narrative foreclosure within the context of chronic disease, enabling early identification of patients at high risk of meaning loss. Next, by integrating individual, psychological, and social facilitators and barriers, predictive models and effective intervention strategies can be developed to reactivate patients' narratives and their capacity to envision a future. Future research should leverage digital technologies and interdisciplinary approaches to shift chronic disease management from a singular biomedical repair paradigm to a collaborative rewriting of patients' life stories. This will holistically improve patients' quality of life and enhance their resilience to disease.

## Data Availability

The original contributions presented in the study are included in the article/Supplementary material, further inquiries can be directed to the corresponding author.
