# Supplementary material for: Narrative foreclosure among patients with chronic diseases: a scoping review
Source: Front Public Health. 2026 Jan 6;13:1735227. doi: 10.3389/fpubh.2025.1735227 (PMC12815864; doi:10.3389/fpubh.2025.1735227)
Supplement: Supplementary file 2 [file Supplementary_file_2.docx]

Appendix 2 Search Details of Literature Search Strategy.

PubMed

| #1 | ((((((((((((((("narrative foreclosure"[Title/Abstract]) OR ("end of story"[Title/Abstract])) OR ("narrative rigidity"[Title/Abstract])) OR ("story closure"[Title/Abstract])) OR ("loss of narrative possibility"[Title/Abstract])) OR ("loss of meaning in life"[Title/Abstract])) OR ("identity stagnation"[Title/Abstract])) OR ("cognitive rigidity"[Title/Abstract])) OR ("existential despair"[Title/Abstract])) OR ("meaning crisis"[Title/Abstract])) OR ("aging stagnation"[Title/Abstract])) OR ("epilogue time"[Title/Abstract])) OR ("static narrative*"[Title/Abstract])) OR ("decline narrative*"[Title/Abstract])) OR ("life story foreclosure"[Title/Abstract])) OR ("rigid interpretation*"[Title/Abstract]) | 269 |
| --- | --- | --- |

Web of Science

| #1 | (((((((((((((((TS=("narrative foreclosure")) OR TS=("end of story")) OR TS=("narrative rigidity")) OR TS=("story closure")) OR TS=("loss of narrative possibility")) OR TS=("loss of meaning in life")) OR TS=("identity stagnation")) OR TS=("cognitive rigidity")) OR TS=("existential despair")) OR TS=("meaning crisis")) OR TS=("aging stagnation")) OR TS=("epilogue time")) OR TS=("static narrative*")) OR TS=("decline narrative*")) OR TS=("life story foreclosure")) OR TS=( "rigid interpretation*") and Preprint Citation Index (Exclude – Database) | 925 |
| --- | --- | --- |

Embase

| #1 | 'narrative foreclosure':ti,ab OR 'end of story':ti,ab OR 'narrative rigidity':ti,ab OR 'story closure':ti,ab OR 'loss of narrative possibility':ti,ab OR 'loss of meaning in life':ti,ab OR 'identity stagnation':ti,ab OR 'cognitive rigidity':ti,ab OR 'existential despair':ti,ab OR 'meaning crisis':ti,ab OR 'aging stagnation':ti,ab OR 'epilogue time':ti,ab OR 'static narrative*':ti,ab OR 'decline narrative*':ti,ab OR 'life story foreclosure':ti,ab OR 'rigid interpretation*':ti,ab | 428 |
| --- | --- | --- |

CINAHL

| #1 | (XB "narrative foreclosure" OR XB "end of story" OR XB "narrative rigidity" OR XB "story closure" OR XB "loss of narrative possibility" OR XB "loss of meaning in life" OR XB "identity stagnation") OR (XB "cognitive rigidity" OR XB "existential despair" OR XB "meaning crisis" OR XB "aging stagnation" OR XB "epilogue time" OR XB "static narrative*" OR XB "decline narrative*") OR (XB "life story foreclosure" OR XB "rigid interpretation*") | 99 |
| --- | --- | --- |

Scopus

| #1 | ( TITLE-ABS-KEY ( "narrative foreclosure" ) OR TITLE-ABS-KEY ( "end of story" ) OR TITLE-ABS-KEY ( "narrative rigidity" ) OR TITLE-ABS-KEY ( "story closure" ) OR TITLE-ABS-KEY ( "loss of narrative possibility" ) OR TITLE-ABS-KEY ( "loss of meaning in life" ) OR TITLE-ABS-KEY ( "identity stagnation" ) OR TITLE-ABS-KEY ( "cognitive rigidity" ) OR TITLE-ABS-KEY ( "existential despair" ) OR TITLE-ABS-KEY ( "meaning crisis" ) OR TITLE-ABS-KEY ( "aging stagnation" ) OR TITLE-ABS-KEY ( "epilogue time" ) OR TITLE-ABS-KEY ( "static narrative*" ) OR TITLE-ABS-KEY ( "decline narrative*" ) OR TITLE-ABS-KEY ( "life story foreclosure" ) OR TITLE-ABS-KEY ( "rigid interpretation*" ) ) | 730 |
| --- | --- | --- |

APA PsycArticles

| #1 | "narrative foreclosure" OR "end of story" OR "narrative rigidity" OR "story closure" OR "loss of narrative possibility" OR "loss of meaning in life" OR "identity stagnation" OR "cognitive rigidity" OR "existential despair" OR "meaning crisis" OR "aging stagnation" OR "epilogue time" OR "static narrative*" OR "decline narrative*" OR "life story foreclosure" OR "rigid interpretation*" | 20 |
| --- | --- | --- |

CNKI

| #1 | SU='narrative foreclosure' OR SU='end of story' OR SU='narrative rigidity' OR SU='story closure' OR SU='loss of narrative possibility' OR SU='loss of meaning in life' OR SU='identity stagnation' OR SU='cognitive rigidity' OR SU='existential despair' OR SU='meaning crisis' OR SU='aging stagnation' OR SU='epilogue time' OR SU='static narrative' OR SU='decline narrative' OR SU='life story foreclosure' OR SU='rigid interpretation' | 114 |
| --- | --- | --- |
